# Myelolipoma of the Pelvis: A Case Report and Review of Literature

**DOI:** 10.3389/fonc.2018.00251

**Published:** 2018-07-03

**Authors:** Seema Sethi, Shivam Thakur, Suzanne Jacques, H. D. Aoun, Paul Tranchida

**Affiliations:** ^1^Department of Pathology, University of Michigan and VA Hospital, Ann Arbor, MI, United States; ^2^Touro College of Osteopathic Medicine, Middletown, NY, United States; ^3^Department of Pathology, Detroit Medical Centre, Wayne State University School of Medicine, Detroit, MI, United States; ^4^Imaging Division, Karmanos Cancer Institute, Wayne State University, Detroit, MI, United States

**Keywords:** Adrenal myelolipoma, extra-adrenal myelolipoma, presacral region, *in situ* hybridization, chromosomal abnormalities

## Abstract

Myelolipomas are uncommon, benign tumors which typically occur in the adrenal glands and consist of mature adipose tissue and benign hematopoietic components. Myelolipomas can occur outside of the adrenal glands, but the presacral region, retroperitoneum, pelvis, and mediastinum are unusual locations for these tumors. It is important to recognize this entity in these locations since they can attain massive sizes leading to pressure symptoms and need to be differentiated from the malignant tumors like liposarcomas. We present a myelolipoma case in the presacral region. Our case illustrates the clinical approach of these tumors in such unusual locations.

## Background

Myelolipomas are a rare entity (1) and, so far, fewer than 50 cases have been reported to have presented as symptomatic (2). Typically, myelolipomas are asymptomatic and unilateral, the adrenal region being the major site, however, many cases have been reported in extra-adrenal regions (3). The second most common site other than the adrenal region is the presacral region, with only a small number of cases reported in perirenal, mediastinum, liver, and stomach locations (3). Histologically, both adrenal and extra-adrenal myelolipomas (EAMs) are similar, and the myeloid component resembles the bone marrow tissue (4). Myelolipomas in extra-adrenal regions are more common in older patients, with higher rates in females than in males (5). The origin of adrenal myelolipoma is not clear but differentiation of either ectopic hematopoietic stem cells or cells of the mesenchyme of the ectopic adrenal tissue has been proposed (3, 6). Although there is no clear consensus on the etiology of adrenal myelolipoma, metaplastic change in the reticuloendothelial cells has been widely accepted, which can be caused by (7, 8) several stimuli such as stress, infection (9, 10), Cushing’s disease (11), hypertension, diabetes, and obesity (12).

Small myelolipomas (<4 cm in diameter) are usually asymptomatic but become symptomatic as the size increases, due to mass effect or hemorrhage (13). Size-related pressure on vital organs can cause back or abdominal pain, high blood pressure, blood in urine, or pain at the site of the tumor. A spontaneous retroperitoneal hemorrhage is one of the well-recognized complication of adrenal myelolipoma (13).

With the diagnostic advancement of radiological tools (ultrasonography, CT, and MRI), most myelolipoma cases are detected incidentally (5). Although CT and MRI can suggest a diagnosis of myelolipoma, these are not conclusive. CT-guided fine-needle aspiration (FNA) of adrenal glands has been shown to provide an accurate diagnosis of myelolipoma (2, 14). FNA in lesions of the adrenal gland provides 90% accurate diagnosis of malignant lesions (15). In large adrenal tumors, an initial diagnosis using FNA may assist in making management decisions regarding whether surgical interventions such as nephrectomy and regional lymphadenectomy can be avoided (16).

With these tumors occurring in unusual locations, such as the presacral region and the retroperitoneum, it is important to clinically recognize them, as they can attain massive sizes and cause pressure symptoms, and need to be differentiated from malignant tumors, including liposarcomas, in those sites. The diagnostic work-up and clinical approach for adrenal myelolipomas is critically important since the subsequent clinical management is dependent upon the diagnosis of this entity. Here, we present a myelolipoma case in the presacral region and emphasize the diagnostic approach of these tumors in such unusual locations. The patient described in this case report provided her written informed consent for its publication.

## Case Presentation

**Figure 1:**
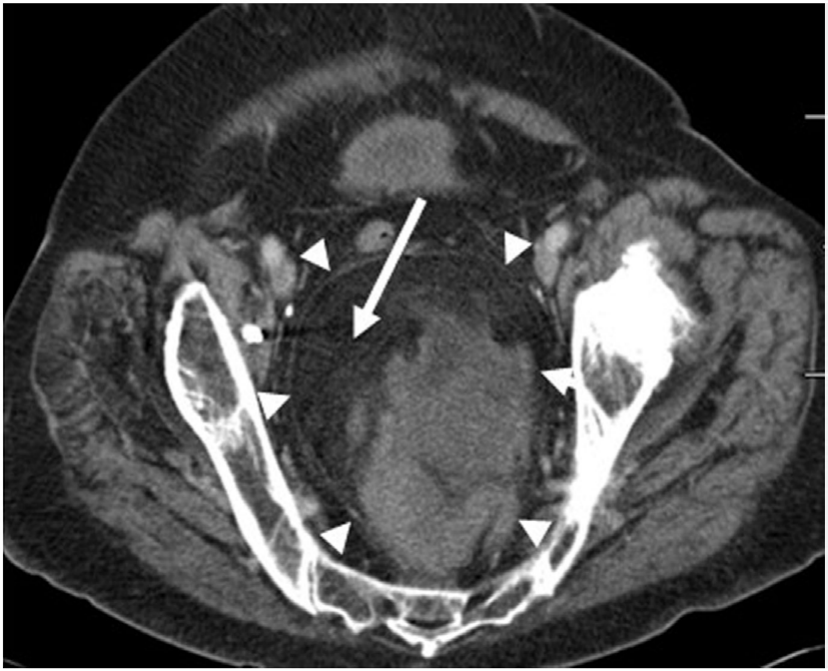
Axial CT image of the pelvis demonstrates a large fat containing mass within the presacral region (arrowheads). Fat within the mass is dark on the CT images (arrow).

**Figure 2:**
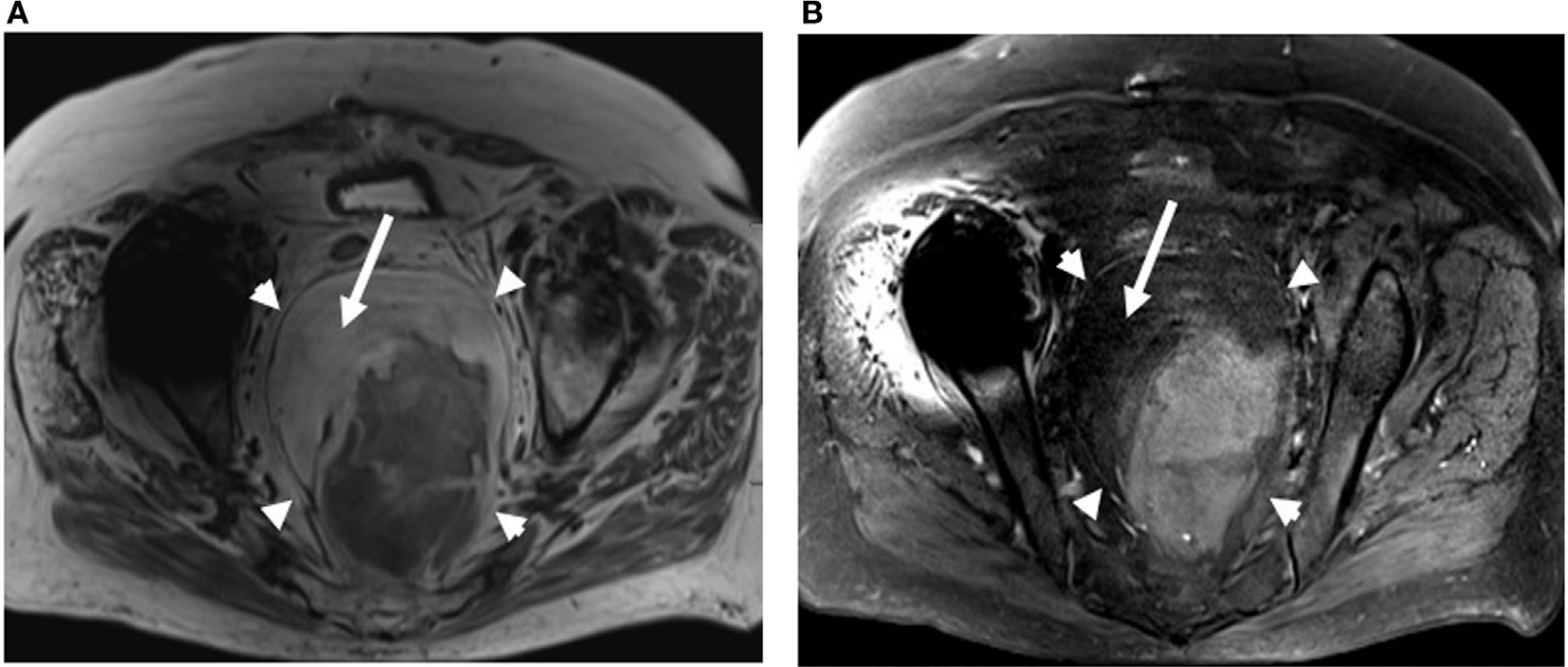
Axial nonfat saturated **(A)** and fat saturated **(B)** T2-weighted MRI images of the pelvis also demonstrates a large fat containing mass within the presacral region (arrowheads). Bright fat on nonfat saturated image **(A)** saturates out on the fat saturated image **(B)** (arrows).

A 70-year-old female patient was admitted with complaints of lower abdominal pain of 5 months duration. Pain was initially intermittent but steadily worsened to require management with narcotics. The patient reported constipation but denied rectal bleeding. At the time of presentation, she had urinary retention that led to the placement of an indwelling Foley catheter which revealed hematuria in the bag. She reported anorexia, nausea, abdominal bloating, and worsening of bilateral leg edema, but did not have any vomiting, hematemesis, chest pain, melena, jaundice, fever, chills, night sweats, or weight loss. Her CT scan showed a large heterogeneous but predominantly fatty pelvic mass compressing the bowel and bladder (**Figure 1**). Two needle core biopsies were done which revealed only benign adipose tissue. Patient was sent for upper and lower gastrointestinal endoscopies and MRI. She further complained of persistent lower abdominal and pelvic pain, and difficulty urinating. An MRI showed a 13 cm × 10 cm × 10 cm pelvic mass that appeared well encapsulated and nested between the rectosigmoid and sacrum. There was no suspicious lymphadenopathy (**Figure 2**). The patient was admitted for the resection of the mass with possible colostomy. She had no history of heart disease, rheumatic fever, neurological disorder, diabetes, ulcers, asthma, tuberculosis, or kidney, liver, or thyroid disease, and had no suspicious lesions on the skin. The patient underwent bilateral ureteral stent placement followed by resection of a large 18 cm sacrococcygeal tumor with en-bloc low anterior rectosigmoid resection and Hartmann’s stump.

On gross examination, the tumor was an unencapsulated, tan-yellow solid mass, measuring 13 cm × 13 cm × 7 cm. The cut surface was tan-yellow, with minute foci of hemorrhage. The tumor was approximately 0.3 cm away from the adjacent colon and did not involve the same.

Histopathologically, the tumor consisted predominantly of mature adipose tissue with no atypia. Few thin and moderately thick collagen bands were noted. Foci of hemorrhage and minute areas of extramedullary hematopoiesis were identified.

Fresh tumor tissue was analyzed for chromosomal abnormalities. Routine karyotyping was done. An abnormal female karyotype was observed after examination of 20 metaphase cells. There was a clonal abnormality: all metaphases had trisomy of chromosome 5. No other abnormality was found. Fluorescence *in situ* hybridization (FISH) analysis of fresh tissue was done, FISH analysis summary is shown in Table 1. LSI DDIT3 and LSI FOXO1 dual color break-apart DNA probes (Vysis Inc.) were used to detect the rearrangements associated with the DDIT3 (CHOP) gene in the 12q13 region and FOXO1 (FKHR) gene in the 13q14 region, respectively. Two hundred interphase cells were examined for each probe. Within the limitations of the procedure, the hybridization produced a normal pattern for both probes, consistent with no translocations, deletions, or rearrangements of the DDIT3 (CHOP)/12q13 or FOXO1 (FKHR)/13q14 genes.

**Table 1:** Fluorescence *in situ* hybridization analysis summary.

Number of DNA probes	4
Number of interphase cells examined	200/probe
Number of metaphase cells examined	NA
Number of cells photographed	4
Number of observers	2
Type of tissue and cultures	Tm(direct)

Fluorescence *in situ* hybridization analysis of formalin-fixed paraffin-embedded tissue was performed using the Vysis MDM2 DNA probe (Abbott Molecular Inc.), which contains two probes. The LSI MDM2 DNA probe labeled spectrum Orange, specific for the MDM2 gene locus on 12q15; while the CEP 12 DNA control probe labeled spectrum Green which is specific for DNA sequence at the centromeric region of chromosome 12p11.1-q11.2. At least 50 non-overlapping cells were scored. The results of hybridization produced an MDM2:CEP12 ratio of 1.0 (Table 2). This was consistent with no amplification of the MDM2 gene, ruling out the possibility of a well-differentiated (WD) liposarcoma. Further immunostaining with HMB-45 and MART-1 was also negative. Based on these findings a final diagnosis of adrenal myelolipoma was rendered.

**Table 2:** Scoring of MDM2 by fluorescence *in situ* hybridization.

MDM2/CEP 12 ratio	1.0
Number of cells examined	100
Average number of MDM2 signals per cell	2.02
Average number of CEP 12 signals per cell	2.02

*Positive: MDM22/CEP12 ratio >2.0*.

*Negative MDM22/CEP12 ratio <2.0*.

Upon recent follow-up (status post-resection 3.5 years), the patient was asymptomatic with no tumor recurrence.

## Discussion

Adrenal myelolipomas are rare tumors comprised of hematopoietic cells and mature adipose tissue (17–19). Hematopoietic elements in myelolipomas have been described as an external marrow (20). Even though adrenal glands are the most common site of occurrence, the incidence of adrenal myelolipoma is very small, ranging from 0.08 to 0.2% mostly in older patients. There are several cases of EAMs in locations such as the presacral region or retroperitoneum which often contain calcifications (20). The typical EAMs are usually asymptomatic, well-defined masses in the abdominal region. Larger EAMs can cause symptoms due to the pressure exerted by a large mass against the surrounding organs, including renal failure due to compression in the region (21–25). Acute hemorrhage related to large myelolipomas is the most noteworthy complication that can present as pain, nausea, vomiting, hypotension, and anemia (8).

A study of 74 patients showed a mean EAM diameter of 8.2 cm with a range from 4 to 15 cm (8, 26). Hemorrhage usually occurs in the larger lesions (>10 cm in diameter) with 89% occurring in males (9).

Diagnosis of EAMs can overlap with other soft tissue tumors, including retroperitoneal lipomas and other tumor types, and, therefore, care must be taken to distinguish it from other soft tissue tumors (8). Radiology may be helpful in suggesting the diagnosis of EAM. Differentiation of adrenal myelolipomas with scant adipose components from other adrenal lesions, including pheochromocytomas, adrenal carcinomas, adrenal metastases, and adrenal lymphomas, should be done by thorough review of the lesion (27).

Although myelolipomas have typical imaging features, these features may overlap with angiomyolipomas, lipomas, teratomas, and liposarcomas (28). Hence, histopathologic examination is important for confirmation of diagnosis. The imaging appearance and pathologic and histologic features of extra-adrenal versus adrenal myelolipomas are quite similar. Several studies have reported that EAMs have two distinct features based on fat content and calcification compared to the adrenal myelolipomas (3, 17, 29). A percutaneous fine-needle biopsy provides a safe and effective tool in making diagnoses when radiological findings are inconclusive. One large study correlated clinical and pathologic patterns of myelolipomas with the CT appearance (8). Correlation was made by size, location, and scoring of CT images for each myelolipoma, and the presence of calcification, hemorrhage, fat, and pseudocapsule (8). The study showed a correlation of pathologic findings with CT findings and concluded that the CT appearance of myelolipomatous foci is different from other adrenal conditions (8).

In surgically documented cases of adrenal myelolipoma, multiple clinical symptoms have been reported, including abdominal pain, palpable tumor, obesity, and hypertension (8). Larger tumors may incur within them hemorrhage, necrosis, calcification, and cyst formation. El-Mekresh et al. reported eight adrenal myelolipoma cases (30) and their associated symptoms. Three cases had hypertension, and one had diabetes mellitus; however, none of the tumors was endocrinologically active. A review of literature reveals that the trisomy of chromosome 5, found on karyotyping of this tumor, is not characteristic for any particular soft tissue tumor. The only tumor characterized as a soft tissue tumor showing trisomy for chromosomes 5 and/or 7 is pigmented villonodular synovitis. The only chromosomal abnormality described was translocation t(3;21) (q25;p11) using conventional cytogenetic techniques, suggesting that myelolipoma is a derivative of misplaced hematopoietic cells (31). Similar chromosomal changes, t(3;21) (q26;p11) were also described in hematopoietic neoplasms. An elaborate study by Bishop et al. (32) used formalin-fixed paraffin-embedded tissue from 19 myelolipoma cases and showed nonrandom X-chromosome inactivation in 8 of 11 female myelolipoma patients suggesting a clonal origin of myelolipomas (32). Supernumerary ring and/or giant chromosome markers at the 12q13-15 region, which includes amplification of MDM2 along with several other genes, are observed in WD and dedifferentiated (DD) liposarcomas (33). In another study in which Pilotti and her group analyzed MDM2 and p53 overexpression in the retroperitoneal WD–DD group (33), almost all (15 of 16 WD and 8 of 8 DD) liposarcomas displayed the MDM2+/TP53+ phenotype. In the non-retroperitoneal WD–DD group, half of the (5 of 11) WD liposarcomas were MDM2+/TP53+ while all DD liposarcomas showed a mutant TP53 phenotype (33). Another study also demonstrated 100% amplification of MDM2 in WD and DD liposarcomas, but no MDM2 amplified in the benign lipomatous lesions. This probe can be a valuable tool in the diagnosis of small biopsy samples of WD lipomatous neoplasms (34).

While myelolipomas are generally asymptomatic and benign, their progressive proliferation may lead to large masses, which can lead to problems associated with pressure against vital organs. Small myelolipomas need clinical and radiologic monitoring, while large myelolipomas that produce unendurable symptoms should incur immediate resection. FISH analysis of MDM2 gene amplification is helpful in differentiating these tumors from WD liposarcomas.

## Ethics Statement

Patient was consented prior to the preparation of Case Report.

## Author Contributions

Data acquisition: SS, SJ, and PT. Data analysis and interpretation: SS, SJ, PT, and ST. Radiological analysis of MRI and CT images: HA. Manuscript preparation: ST and SS.

## Conflict of Interest Statement

The authors declare that the research was conducted in the absence of any commercial or financial relationships that could be construed as a potential conflict of interest.
